# Preclinical Detection of Prions in Blood of Nonhuman Primates Infected with Variant Creutzfeldt-Jakob Disease 

**DOI:** 10.3201/eid2601.181423

**Published:** 2020-01

**Authors:** Luis Concha-Marambio, Marcelo A. Chacon, Claudio Soto

**Affiliations:** University of Texas, Houston, Texas, USA (L. Concha-Marambio, M.A. Chacon, C. Soto);; Universidad de los Andes, Santiago, Chile (L. Concha-Marambio, C. Soto)

**Keywords:** prions, variant Creutzfeldt-Jakob disease, protein misfolding cyclic amplification, diagnosis, blood, nonhuman primates, vCJD

## Abstract

Variant Creutzfeldt-Jakob disease (vCJD) is caused by prion infection with bovine spongiform encephalopathy and can be transmitted by blood transfusion. Protein misfolding cyclic amplification (PMCA) can detect prions in blood from vCJD patients with 100% sensitivity and specificity. To determine whether PMCA enables prion detection in blood during the preclinical stage of infection, we performed a blind study using blood samples longitudinally collected from 28 control macaques and 3 macaques peripherally infected with vCJD. Our results demonstrate that PMCA consistently detected prions in blood during the entire preclinical stage in all infected macaques, without false positives from noninfected animals, when using the optimized conditions for amplification of macaque prions. Strikingly, prions were detected as early as 2 months postinoculation (>750 days before disease onset). These findings suggest that PMCA has the potential to detect vCJD prions in blood from asymptomatic carriers during the preclinical phase of the disease.

Prion diseases are rare and fatal neurodegenerative diseases transmitted by infectious proteinaceous agents called prions, which are composed of a disease-associated misfolded version (PrP^Sc^) of the normally expressed prion protein (PrP^C^) (*1**–**3*). Prion diseases affect humans and various species of mammals, including cattle, sheep and goats, and cervids (*4*). In humans, Creutzfeldt-Jakob disease (CJD) is the most common prion disease and can be sporadic (sCJD), familial (fCJD), or iatrogenically transmitted (iCJD). In the 1990s, a new variant of CJD (vCJD) was described in the United Kingdom (*5*); this variant is a result of interspecies transmission of bovine spongiform encephalopathy (BSE) prions from cattle to humans (*6**–**8*). Unlike classical CJD, vCJD presents an extensive peripheral deposition, with demonstrated PrP^Sc^ accumulation in various peripheral tissues, particularly lymphoreticular tissues (spleen, appendix, and tonsil) (*9**–**11*). vCJD has been suggested to be transmitted among humans by transfusion of nonleukodepleted erythrocytes or purified protein factors from plasma (*12**,**13*). A study performed in transgenic mice models to compare the risk for primary and secondary transmission of vCJD showed that, although transmission of BSE to humans is probably restricted by the presence of a major species barrier, secondary transmission between humans has a substantially reduced barrier (*14*). Moreover, this study showed that all humans, irrespective of PrP codon-129 genotype, could be susceptible to secondary transmission of vCJD through routes such as blood transfusion. A lengthy preclinical disease is predicted by these models, which may represent a risk for further disease transmission (*14*).

Detection of prions in blood has been hampered because of the unconventional nature of prions (absence of nucleic acids) and the minute amount of them circulating in blood, making them difficult to detect even by bioassay in transgenic mice (*15*). Extraction protocols to enrich PrP^Sc^ from blood have been developed and coupled to antibody detection methods (*16*), but sensitivity was only 70% for end-stage disease blood samples (*17*). In contrast with conventional biochemical methods, we developed a detection platform for self-replicating PrP^Sc^ called protein misfolding cyclic amplification (PMCA) (*18*). During PMCA, small amounts of infectious PrP^Sc^ aggregates convert PrP^C^ into PrP^Sc^, producing larger protein aggregates that are fragmented into many smaller nucleating seeds for the continued in vitro conversion of PrP^C^ into PrP^Sc^ (*18**–**20*). This elongation/fragmentation process is performed cyclically to exponentially amplify PrP^Sc^, facilitating its detection. PMCA can amplify vCJD prions from brain homogenate (BH) diluted 10^−10^- to 10^−11^-fold, reaching a 10–100 billion-fold amplification (*21*). This level of amplification has allowed detection of prions in blood and urine samples from vCJD patients (*21**,**22*), reaching sensitivities and specificities approaching 100% in experiments confirmed in various laboratories (*23**,**24*).

It is unclear how early prions can be detected in the blood of infected persons at the preclinical stage of the disease. In this study, we analyzed the preclinical detection of prions in blood samples from macaques (*Macaca fascicularis*) experimentally infected with the vCJD agent as an animal model for infected asymptomatic human carriers.

## Materials and Methods

### Nonhuman Primate Experimental Infection and Longitudinal Blood Collection

Experimental inoculation of macaques and collection of blood materials was done at the Food and Drug Administration (FDA) laboratory (Silver Spring, Maryland, USA) as previously described (*25*). In brief, macaque-adapted vCJD (m-vCJD) was generated by intracerebral injection with BH from a confirmed vCJD patient. A 10% BH solution from the terminally ill macaque was used for intraperitoneal (2 mL) and intravenous (1 mL) inoculation into the 3 macaques used in this study. Blood samples were collected every 2 months for the first year and every month for the rest of the experiment. Samples were collected in either citrate phosphate dextrose buffer or EDTA. Part of the blood was separated to prepare plasma, buffy coat (BC), and erythrocyte components. We received panels of deidentified samples for blind experiments (Appendix).

### Processing of Blood Samples

We previously described a sarkosyl precipitation method to extract vCJD prions from blood and remove interferences in the PMCA assay (*21*). In brief, we incubated 250 or 500 μL of blood or blood fractions with an equal volume of 20% sarkosyl for 10 min at room temperature. We then ultracentrifuged the mixture at 100,000 × *g* for 1 h at 4°C. After washing the pellet, we resuspended it in PMCA substrate for subsequent amplification and detection. Although this procedure might be difficult to implement for routine testing of blood samples, we have previously shown that processing and centrifugation may be overcome by working with a smaller volume of blood samples (*21*).

### PMCA Protocol

The PMCA protocol for amplification of human prions has been described elsewhere (*20**–**22*), although some modifications were made for the amplification of preclinical samples (described later). As PMCA substrate, we used 10% BH from transgenic mice expressing human PrP^C^ with methionine/methionine at codon 129 (TgHu129M). These mice express PrP at 16-fold the levels of expression of endogenous protein. We prepared BH in conversion buffer (PBS supplemented with 150 mmol/L NaCl and 1% TritonX-100) with protease inhibitors (complete, EDTA-free; Roche, https://www.roche.com). After performing homogenization, we removed debris by centrifugation at 800 × *g* at 4°C for 1 min. We vortexed, aliquoted, and stored the supernatant at −80°C until use. We supplemented the homogenate with 0.05% digitonin and 12 mmol/L EDTA; in some cases we also added 100 μg/mL of heparin as indicated. We placed samples in 0.2 mL PCR tubes (Eppendorf, https://www.eppendorf.com) containing 3 polytetrafluoroethylene beads (Hoover Precision Bioproducts, http://www.hooverprecision.com) and sonicated them for 30 s every 30 min in a microplate sonicator (QSonica Q700*,* https://www.sonicator.com), using a titanium horn. When we amplified blood and blood fractions, the first round of PMCA included 144 cycles followed by subsequent rounds of 96 cycles, unless otherwise specified. After a PMCA round, we started a new PMCA round by adding 10 μL of each sample to new PCR tubes containing 3 beads and 90 μL of fresh substrate. When analyzing BC samples, we made a pseudo-passage of the first round, in which we added 90 μL of fresh substrate to 100 μL from the first round with no dilution of the material, to reduce the viscosity of the solution by adding more reaction mixture containing substrate. We used diluted samples of vCJD BH as positive controls. We prepared this material from the frontal cortex of a human with pathology-confirmed vCJD.

### Proteinase K Digestion and Western Blotting

After PMCA, we digested all samples using proteinase K (PK) at a concentration of 50 μg/mL for 1 h at 37°C. We stopped PK digestion by boiling the sample at 100°C for 10 min after mixing with NuPage or Novex sample loading buffer (NuPage Bis-Tris gels with MES buffer and Novex Tris-Glycine gels with Tris-SDS buffers). We transferred proteins onto nitrocellulose membranes (0.45 μm; Amersham Biosciences, https://www.gelifesciences.com) and probed them with monoclonal antibody 6D11 (1:20,000) for 1 h at room temperature, while we used secondary anti–mouse antibody (Sigma, https://www.sigmaaldrich.com) at 1:3,000 dilution and incubated for 1 h. We used ECL chemioluminiscent reagent (Amersham) and a Chemidoc imaging system (BioRad, https://www.bio-rad.com) to develop and capture the images.

## Results

### m-vCJD Prion Conversion of Human PrP^C^ into PrP^Sc^ in PMCA

**Figure 1:**
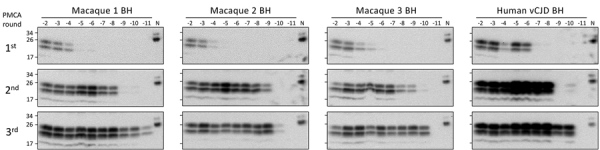
Amplification of macaque-adapted vCJD prions by PMCA. BH from 3 macaques peripherally infected with macaque-adapted vCJD was serially diluted and amplified by 3 rounds of PMCA, using BH from transgenic mice expressing human normally expressed prion protein with methionine at codon 129 (TgHu129M) as substrate. Human BH from a vCJD patient was analyzed as positive control. After completion of the 3 rounds of PMCA, samples were digested with 50 μg/mL of proteinase K and analyzed by Novex SDS-PAGE (https://www.thermofisher.com). N refers to transgenic mouse normal BH without proteinase K treatment, which was used as a migration control. BH, brain homogenate; PMCA, protein misfolding cyclic amplification; vCJD, variant Creutzfeldt-Jakob disease.

We previously showed that PMCA can efficiently amplify vCJD prions using TgHu-PrP^C^ substrate (*21*). Because the sequence of macaque and human PrP has 9 aa differences, we first evaluated whether human PrP^C^ could be converted into PrP^Sc^ in PMCA by m-vCJD prions. Therefore, we prepared 10-fold serial dilutions of BH from the 3 macaques peripherally infected with the vCJD agent and analyzed them with 3 rounds of PMCA, alongside similar dilutions of BH from a human with confirmed vCJD (Figure 1). In the first round of PMCA, the detection limit in the macaque BHs was 10^−4^ to 10^−5^, whereas human vCJD was detectable up to a dilution of 10^−6^. In the second round, we detected vCJD and m-vCJD BH at 10^−9^ to 10^−10^ dilutions, and in the third round the detection limit decreased to 10^−10^ or 10^−11^ depending on the macaque. In summary, 3 rounds of PMCA were necessary to amplify prions in m-vCJD BH dilutions to similar levels as prions in human vCJD BH dilutions, albeit with a reduced conversion efficiency in the first round.

### Detection of m-vCJD Prions by PMCA from Blood Collected at Final Bleed

**Figure 2:**
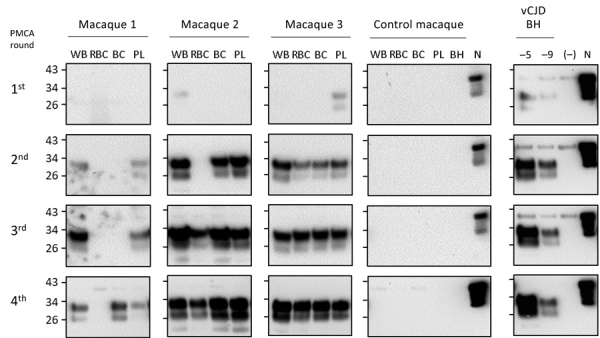
Detection of macaque-adapted vCJD prions in blood and blood fractions of macaques collected at the final bleed. WB, RBC, BC, and PL collected at the terminal bleed of 3 macaques infected with macaque-adapted vCJD and 1 noninfected control were processed and analyzed by 4 PMCA rounds. Dilutions of vCJD BH of 10^−5^ (−5) and 10^−9^ (−9) were analyzed as positive controls; an unseeded reaction (−) was used as a negative control. After completion of the 4 rounds of PMCA, samples were digested with 50 μg/mL of proteinase K and analyzed by Western blot. N refers to transgenic mouse normal BH without proteinase K treatment, which was used as a migration control. BC, buffy coat; BH, brain homogenate; PL, plasma; PMCA, protein misfolding cyclic amplification; RBC, erythrocytes; vCJD, variant Creutzfeldt-Jakob disease; WB, whole blood.

We wanted to determine whether endogenous m-vCJD prions in blood could be detected using the current PMCA conditions. We processed whole blood, plasma, BC, and erythrocyte samples by using sarkosyl precipitation and analyzed them with 4 rounds of PMCA (Figure 2). Similar to our previous report in humans, prions in the m-vCJD whole blood samples were detected by PMCA in the second round, whereas m-vCJD erythrocytes displayed lower amplification with 1 of 3 samples remaining negative after 4 PMCA rounds. Prions in m-vCJD plasma and BC samples were readily detectable by the second round in 2 of 3 infected macaques. As expected, whole blood, blood fractions, and BH from a control macaque were all negative after 4 rounds of PMCA.

**Figure 3:**
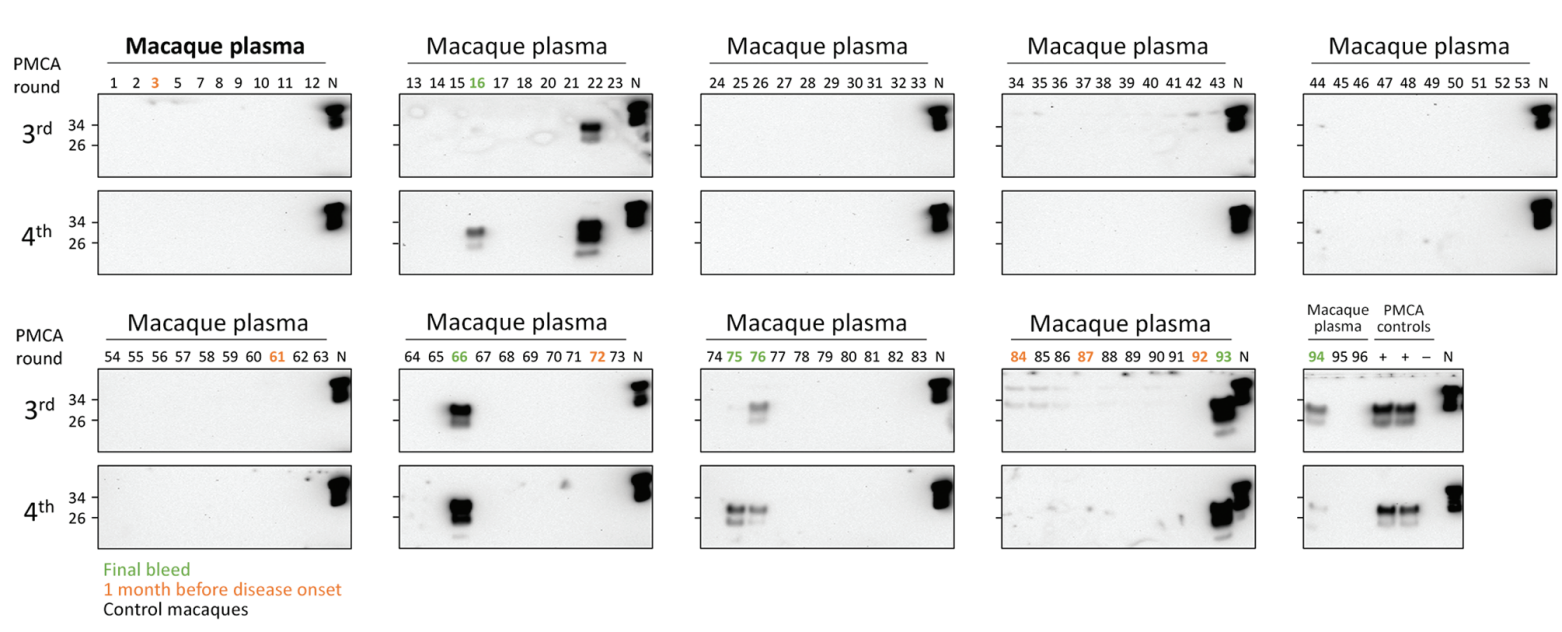
PMCA analysis of deidentified plasma samples from macaques infected with macaque-adapted vCJD and control macaques. Plasma samples from 2 infected (M1 and M3) and 28 control macaques were sarkosyl precipitated and analyzed by 4 rounds of PMCA. This panel of samples included 6 plasma samples collected at the final bleed (M1, #16, #75, #76; M3, #66, #93, #94), 6 plasma samples collected 1 month before disease onset (M1, #72, #84, #92; M3, #3, #61, #87), and 81 plasma samples from control macaques (93 samples total). Tubes with samples #4, #6, and #19 were partially or totally open upon arrival and were not analyzed. Dilutions of vCJD BH of 10^−5^ and 10^−9^ were used as a positive control; the negative control was the unseeded reaction. After completion of the 4 rounds of PMCA, samples from the third and fourth rounds were digested with 50 μg/mL of proteinase K and then analyzed by Western blot. N refers to transgenic mouse normal BH without proteinase K treatment used as a migration control. BH, brain homogenate; PMCA, protein misfolding cyclic amplification; vCJD, variant Creutzfeldt-Jakob disease.

To determine the reproducibility and stability of these samples, we analyzed a panel of 50 deidentified plasma and whole blood samples from infected and control macaques that were subjected to 1–6 freeze/thaw cycles. Using plasma, we detected 12 of 12 samples from infected macaques, whereas only 9 of 12 whole blood samples were found positive (data not shown). Next, we analyzed an additional panel of 93 blinded plasma samples from 28 control and 2 m-vCJD infected macaques, including terminal bleed samples and samples collected 1 month before clinical signs (Figure 3; Appendix Table 1). The panel included 96 samples, but 3 tubes were partially or totally open upon delivery and were excluded from the study, while keeping identification numbers provided by FDA for the rest of the blinded samples (1–96). After 4 rounds of PMCA, we detected prions in 3 of 3 replicates from 2 m-vCJD samples collected at the final bleed; all the controls were negative in all 3 replicates, except for 1 macaque that was negative in 2 of 3 replicates (Appendix Table 1). However, under these conditions, we were unable to detect m-vCJD preclinical samples. Therefore, the sensitivity of this PMCA setting was not sufficient for preclinical detection of m-vCJD prions in blood.

### Standardization of PMCA for Preclinical Detection of Prions in Blood Samples

**Figure 4:**
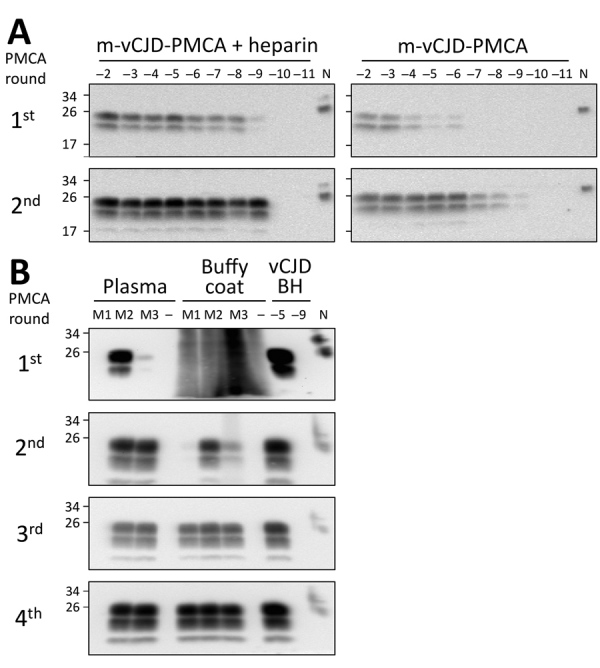
PMCA optimization for detection of macaque-adapted vCJD (m-vCJD) prions in preclinical blood samples. A) Tenfold serial dilutions of m-vCJD BH were amplified by the regular PMCA substrate (right panel) and substrate supplemented with 100 μg/mL heparin (left panel). After completion of 2 PMCA rounds, samples were digested with 50 μg/mL of PK and analyzed by Western blot. B) PL and BC (500 μL) samples collected 1 month before disease onset from 3 m-vCJD infected macaques (M1, M2, and M3) were sarkosyl precipitated and analyzed by 4 PMCA rounds using hep-substrate. Samples from noninfected macaques were analyzed as negative controls (–). N refers to transgenic mouse normal BH without proteinase K treatment, which was used as a migration control. BH, brain homogenate; PMCA, protein misfolding cyclic amplification; vCJD, variant Creutzfeldt-Jakob disease.

The limiting factor for prion amplification using blood samples was probably the conversion inefficiency during the first PMCA round. During this round, m-vCJD prions needed to overcome a small species barrier and natural PMCA inhibitors remaining from blood. Because heparin has been shown to boost the in vitro replication of human prions (*26*), we studied the effect of heparin on the replication of brain m-vCJD prions, using human PrP^C^ as substrate (Figure 4, panel A). Addition of heparin to the PMCA substrate (hep-substrate) enhanced the replication of m-vCJD prions by 3 orders of magnitude in a single round of 96 cycles. Moreover, the amount of PrP^res^ detected after PK digestion by Western blot was clearly higher with hep-substrate, which also decreases the chance of false negatives. Therefore, we used enhanced PMCA with hep-substrate for detection of m-vCJD prions in blood and blood fractions collected at the final bleed. This modification allowed detection in most samples after only 1 round of PMCA (data not shown). However, we could detect prions in only 1 of 3 preclinical plasma samples, although the positive sample required only 1 PMCA round of 144 cycles (data not shown). The amount of PrP^Sc^ in blood at the preclinical stage of the disease is probably very low; thus, we increased the sample volume from 100 μL to 500 μL and compared the detection of prions in preclinical m-vCJD plasma and BC (Figure 4, panel B). Three rounds of PMCA allowed detection of prions in all 3 preclinical m-vCJD BC samples, whereas only 2 m-vCJD plasma samples were positive after 4 PMCA rounds. This is not entirely surprising, because it has been extensively reported that the largest concentration of prions in blood is located in the BC fraction (*27**,**28*). Therefore, given the expected higher concentration of prions in BC and availability of samples, we decided to use BC for the experiments of preclinical detection.

### Prion Detection with High Specificity and Sensitivity Throughout Preclinical Stage

**Figure 5:**
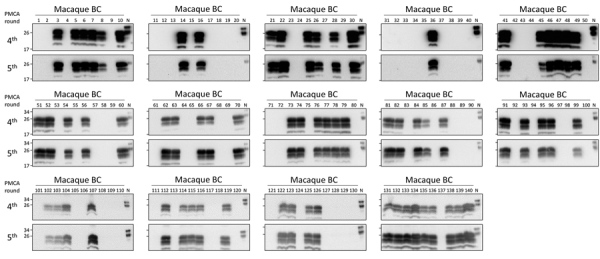
Preclinical detection of macaque-adapted vCJD prions in BC of peripherally infected macaques. A total of 140 deidentified samples (500 μL each) were sarkosyl precipitated and analyzed by 5 rounds of PMCA. After amplification, samples from the fourth and fifth rounds were digested with 50 μg/mL of PK and analyzed by Western blot. N refers to transgenic mouse normal BH without proteinase K treatment, which was used as a migration control. BH, brain homogenate; m-vCJD, macaque-adapted vCJD; PMCA, protein misfolding cyclic amplification; vCJD, variant Creutzfeldt-Jakob disease.

**Figure 6:**
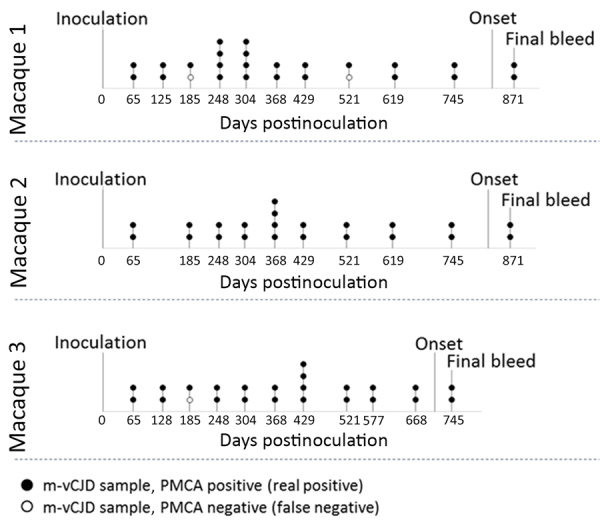
Schematic representation of the animals and samples used in study of preclinical detection of prions in blood of nonhuman primates infected with vCJD. The 72 m-vCJD samples previously analyzed by PMCA (Figure 5) were collected throughout the whole incubation period, starting 65 dpi until the final bleed. The first blood collection at 65 days postinoculation represents 759 (M1 and M2) and 644 (M3) days before the onset of the first neurologic signs. The 72 m-vCJD BC samples included 28 duplicates (represented as 2 circles in the timeline) and 4 quadruplicates (represented as 4 circles in the timeline). Open circles represent m-vCJD BC samples that were PMCA negative; dark circles represent m-vCJD BC samples that were PMCA positive. BH, brain homogenate; m-vCJD, macaque-adapted vCJD; PMCA, protein misfolding cyclic amplification; vCJD, variant Creutzfeldt-Jakob disease.

Using enhanced-PMCA and BC samples, we performed a comprehensive study of PrP^Sc^ detection in longitudinally collected blood samples from macaques peripherally infected with vCJD. The goal was to estimate sensitivity and specificity, as well as the earliest time point in which prions can be consistently detected in blood. Using 5 rounds of enhanced PMCA, we analyzed 140 blinded BC samples either collected from 23 uninfected macaques (Appendix Table 2) or the 3 m-vCJD challenged macaques throughout the entire length of infection; we analyzed 29 samples collected from 3 macaques in duplicate or quadruplicate (Appendix Tables 3–5). BC samples were heavily contaminated with erythrocytes, making them highly viscous. In turn, the pellets from these samples were consistently larger than the previous BC samples, which resulted in many PMCA reactions forming a paste that could not be pipetted to seed the second PMCA round. To work around this issue, we further modified the PMCA protocol by incorporating a pseudo-passage, in which we added 90 μL of hep-substrate to the first round of PMCA and performed amplification cycles for 2 more days. Subsequently, we performed 4 regular PMCA rounds; samples from the fourth and fifth rounds were PK digested and analyzed by Western blot (Figure 5). Of the 140 BC samples, we found 69 positives by PMCA from the 72 m-vCJD positive samples, whereas all the 68 controls were found negative. When we grouped all the replicates of each collected sample, our results showed that all collected samples from m-vCJD macaques have >1 positive signal. The m-vCJD BC replicates that were negative (empty circles in Figure 6) did not correlate with earlier preclinical times, suggesting that these replicates were negative because of the quality of the sample rather than a particularly low concentration of prions that was below the detection limit. It is noteworthy that the 3 negative samples were labeled as having qualitative differences before analyzing the results (2 out of 3 were unusually viscous during sarkosyl precipitation, and the other had a unique colorless pellet). Because the specificity of PMCA is extremely high and we expect the quantity of PrP^Sc^ in blood at the preclinical phase of the disease to be low, we defined the collected samples as positive if >1 replicates are positive. Therefore, we consistently detected prions from all 3 macaques throughout the entire incubation period, starting from the first blood collection at 65 days postinoculation (dpi) until the final bleed. Thus, preclinical detection was achieved 759 days before onset (dbo) for macaques 1 and 2 and 644 dbo for macaque 3 (Figure 6). Considering all 140 samples separately, the detection of m-vCJD prions in BC samples from the preclinical panel reached a sensitivity of 95.8% (95% CI 88.3–99.1%) and a specificity of 100% (95% CI 94.7–100%). However, when grouping the replicates of each collected sample, sensitivity and specificity were 100%.

### Prions in Very Early Stages as Endogenously Generated m-vCJD Prions, Not Part of Inoculum

**Figure 7:**
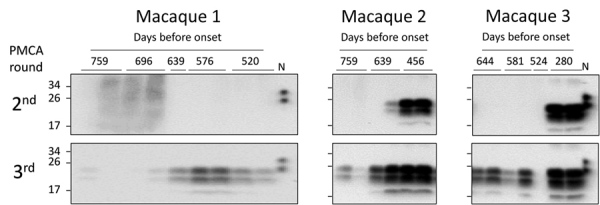
Detection of m-vCJD prions by PMCA in macaques during early stages of disease. These prions were probably endogenously generated rather than present in the inoculum. The second and third rounds of the PMCA-positive preclinical buffy coat samples were digested with 50 μg/mL of proteinase K and analyzed by Western blot. Samples were arranged from the earliest preclinical on the left to the closest to disease onset on the right. N refers to transgenic mouse normal BH without proteinase K treatment, which was used as a migration control. BH, brain homogenate; m-vCJD, macaque-adapted vCJD; PMCA, protein misfolding cyclic amplification; vCJD, variant Creutzfeldt-Jakob disease.

Given the peripheral infection route used in this bioassay and the very early detection achieved by PMCA, there was a possibility of PMCA detecting the inoculum. This result is unlikely, however, because we have shown previously in rodent models that the half-life of radiolabeled PrP^Sc^ in blood is 3.24 h, and <1% of the injected dose remains detectable after 24 h (*29**,**30*). If the clearance rate is the same in macaques, the injected material should be eliminated from blood at a rate of 2 logs per day. Therefore, after 6 days the amount of inoculum remaining in blood would be equivalent to a 10^−12^ dilution of m-vCJD BH. Thus, it is highly unlikely that the early detection is explained by the presence of the inoculum in blood 2 months after injection. Nevertheless, to shed light on this issue, we analyzed the second and third rounds of the PMCA-positive preclinical BC samples by Western blot (Figure 7), considering that we previously established that the number of rounds needed to detect a sample as positive correlates with the amount of prions in the sample (*31*). In macaque 1, all samples were negative in the second round of PMCA. In the third round, samples collected 65 dpi were negative, whereas consistent positive signals started appearing 128 dpi (639 dbo). In macaques 2 and 3, BC samples collected closest to the inoculation time were negative in the second round of PMCA, whereas consistent detection started at 368 dpi (456 dbo) for macaque 2 and 429 dpi (280 dbo) for macaque 3. These results show that samples collected closer to the inoculation time were negative in initial PMCA rounds, indicating lower quantity of PrP^Sc^. Conversely, samples collected at later times were positive in the second round, suggesting higher amounts of PrP^Sc^ in blood. These data allude to a buildup of prions in blood during the incubation period, suggesting that we are not detecting the inoculum.

## Discussion

The future of the vCJD epidemic is still uncertain, with recent estimations of 1 carrier of prion infection in every 2,000 persons who lived in the United Kingdom during the BSE epidemic (*32*). However, in a recent update from the same group, prions were detected in a cohort of persons born after the BSE epidemic, suggesting that the number of silent carriers of prions might be higher than originally anticipated. Before 2017, the population at risk was believed to be restricted to persons carrying Met-Met at codon 129 in the *PRNP* gene, because all clinical vCJD cases occurred in 129 methionine homozygotes (129MM). However, the confirmation of the first patient heterozygous for the *PRNP* codon 129 (129MV) has altered that perspective (*33*). Therefore, 89% of the UK population (42% 129MM and 47% 129MV) exposed to BSE are potential carriers who could harbor prions in their peripheral organs and blood. The likely transmission of vCJD through blood components has been reported and represents a risk for iatrogenic transmission of this disease (*11**–**13**,**34*). Although the policies implemented to control the BSE epidemic have contributed to the decline of vCJD cases (*35*), the number of persons silently carrying infectious prions in their peripheral organs and fluids is unknown. Therefore, a highly sensitive and specific detection method for prions is needed to screen the blood supply to ensure its safety.

The recent demonstration that PMCA enables the accurate detection of prions in blood of confirmed vCJD patients was a major milestone in the quest for a vCJD blood test. Independent studies from us and another group obtained a 100% specificity and sensitivity for vCJD prion detection in blood during the clinical stage of the disease (*21**,**24*). Given that the incubation period for some human prion diseases can be >50 years (*36*), a test to screen blood needs to detect prions as early as possible during the asymptomatic stage of the disease. To analyze the efficacy of PMCA for preclinical detection of vCJD prions in blood, we used samples longitudinally collected throughout the incubation period from nonhuman primates that were deliberately infected with vCJD prions. The study included samples collected >2 years before the first neurologic symptoms and as little as 2 months after animals were infected. Considering each replicate individually, sensitivity of the assay was 96% and specificity of the assay was 100%. Considering individual animals (samples collected in duplicates and quadruplicates), sensitivity and specificity both reached 100%. Macaque 1 showed lower levels of PrP^Sc^ in blood (Figures 2; 4, panel B; 7), despite the indistinguishable disease progression described by McDowell et al. between macaques 1 and 2 (*25*). The difference in PrP^Sc^ levels in blood is probably the result of intrinsic animal-to-animal variability, but it could be an indication that PrP^Sc^ levels in blood and brain are independent, given that all 3 macaques showed similar levels of PrP^Sc^ in the brain. It could also suggest differences in the clearance of PrP^Sc^ from brain to blood, perhaps indicating changes in blood–brain barrier tightness. The differences may also suggest different levels of peripheral prion replication. At this time we do not have enough information to distinguish among these possibilities.

Our results confirm and extend a previous report by Lacroux et al., who used a similar model in macaques infected with vCJD in which they detected prions 960 and 990 days before onset of the disease in 2 macaques, which showed clinical signs 43–46 months postinoculation (*23*). Given that we used an animal model experimentally infected with vCJD-BH, we cannot necessarily conclude that similar detection levels will be obtained in human samples. In addition, PrP^Sc^ detection does not necessarily indicate that the material would be infectious in vivo, considering that PMCA is orders of magnitude more sensitive than the infectivity bioassay (*31*). This finding raises a difficult ethical issue of how to deal with persons who return a PMCA-positive blood test, especially considering that no treatment is available for this disease.

Unfortunately, few blood samples collected before vCJD developed in donors are available to test PMCA for preclinical detection in humans. In a previous study, researchers analyzed such samples from 2 donors and found them positive for PMCA (*24*). Overall, our results suggest that PMCA has the potential to be used as a screening method to improve the safety of the blood supply and perhaps as a tool to determine the prevalence of prion carriers in countries at high risk for vCJD (e.g., United Kingdom and France). Future studies should aim to confirm the high sensitivity and specificity of the assay using many human control samples and an alternative model for preclinical detection in blood, such as sheep transfused with blood from BSE-infected sheep. It will also be crucial to test all available samples from persons affected by vCJD who donated blood before the disease appeared. Finally, it is necessary to highlight that the principles behind PMCA may be also used to detect misfolded protein aggregates responsible for common neurodegenerative diseases, such as Alzheimer’s and Parkinson’s diseases, which also self-propagate by a prionlike seeding mechanism (*37**,**38*). We and others have shown that seeding amplification assays can be implemented to detect misfolded aggregates composed of amyloid-β, tau, and α-synuclein in human biologic fluids (*39**–**44*), suggesting that PMCA represents a platform technology for highly sensitive detection of misfolded proteins.

Appendix Additional information about preclinical detection of prions in blood of nonhuman primates infected with variant Creutzfeldt-Jakob disease.
