# Supplementary material for: Preclinical Detection of Prions in Blood of Nonhuman Primates Infected with Variant Creutzfeldt-Jakob Disease
Source: Emerg Infect Dis. 2020 Jan;26(1):34–43. doi: 10.3201/eid2601.181423 (PMC6924915; doi:10.3201/eid2601.181423)
Supplement: Appendix — Additional information about preclinical detection of prions in blood of nonhuman primates infected with variant Creutzfeldt-Jakob disease. [file 18-1423-Techapp-s1.pdf]

Article DOI: https://doi.org/10.3201/eid2601.181423

# Preclinical Detection of Prions in Blood of Nonhuman Primates Infected with Variant Creutzfeldt-Jakob Disease

## Appendix

### Genotyping of Hu-PrP Mice

For a PMCA substrate we used 10% BH from transgenic mice expressing human $PrP^C$ with methionine/methionine at codon 129 (TgHu129M, donated by Dr. Glenn Telling, Colorado State University). These mice express PrP at 16 times the levels of expression of endogenous protein. For genotyping, tails were obtained by anesthetizing the animal using isoflurane mixed with oxygen. We cut a tail segment 0.3–0.5 mm long with hot scissors and placed it in a DNase-free tube. Scissors were dipped in bleach and flamed to remove DNA between animals. We extracted DNA by incubating the tails with 100 µL Tissue Extraction Solution (Sigma, cat. no. E7526, https://www.sigmaaldrich.com) and 25 µL Tissue Preparation Solution (Sigma, cat. no. T3073) for 1 h at room temperature. Then we boiled the samples at 100°C for 3 min after which we added 100 µL of Neutralization Solution (Sigma, cat. no. N3910). For the PCR reaction, each reaction tube contained 0.5µL of each primer, 7.5 µL Taq polymerase (NEB, cat. no. M0270S, https://www.neb.com), 6 µL water and 1 µL of extracted DNA. PCR consisted of 35 cycles, each one including 30s at 95°C, 30s at 62°C, 30s at 68°C. Primers used were Php PRP5′: GAACTGAACCATTTCAACCGA. PhpPRP3′: AGAGCTACAGGTGGATAA CC.

### Brain Collection for PMCA

Mice were euthanized using low $CO_2$ concentration, to avoid animal suffering, according to the animal protocol approved by the Center for Laboratory Animal Medicine and Care and the animal welfare committee of the University of Texas Health Science Center at Houston (UTHealth). After making sure the animal was dead, we exposed the heart. The surface of the

atrium (upper and darker part of the heart) was punctured and 50 mL of cold infusion buffer (1X PBS (GE Healthcare Life Sciences, cat. no. SH30256.02, https://www.gelifesciences.com) + 5 mM EDTA (Invitrogen, cat. no. 15575020, https://www.thermofisher.com) were injected at the ventricle. Another 50 mL were injected if blood was still coming out the atrium. Once clear buffer came out of the atrium, the brain was removed, rinsed in perfusion buffer, and snap-frozen in liquid nitrogen ($LN_2$). Frozen brains were stored at –80°C until use.

## Substrate Preparation

Brains were homogenized on ice in cold conversion buffer (1X PBS [GE Healthcare Life Sciences, cat. no. SH30256.02] supplemented with 150 mM NaCl and 1% TritonX-100) with protease inhibitors (complete, EDTA-free [Sigma, cat. no. 4693132001]) at 10% (wt/vol) using a Potter-Elvehjem homogenizer. After homogenization, tissue debris was removed by centrifugation at 800 × *g* and 4°C for 1 min. The pellet was discarded, the supernatant was placed on ice, mixed by vortexing, and aliquoted into 1.5 mL RNase-free microcentrifuge tubes (Eppendorf, cat. no. 022431081, https://www.eppendorf.com). The brain homogenate (BH) was then snap-frozen in $LN_2$ and stored at –80°C until use. We strongly recommend thawing BH aliquots only once (prepare single-use aliquots). Freeze–thaw cycles will sharply decrease the amplification efficiency. Before starting the PMCA procedure, the homogenate was supplemented with 0.05% digitonin (Sigma, cat. no. D5628) and 12 mM EDTA (Invitrogen, cat. no. 15575020); in some cases, we also added 100 μg/mL of heparin (Sigma, cat. no. H3393–50KU) as indicated.

## Sarkosyl Precipitation of Prions

All the PMCA work including vCJD samples was performed in a biosafety cabinet class II type B2 on a biosafety level 3 (BSL3) facility. A total of 250–500 μL of whole blood (WB), plasma (PL), or buffy coat (BC) was mixed with equal volume of 20% sarkosyl (Fisher BioReagents, cat. no. BP234–500, https://www.fishersci.com) prepared in water. This mixture was incubated in an end-over-end mixer at room temperature for 10 min and ultracentrifuged at 100,000 × *g* for 1h at 4°C. The pellet was washed with 500 μL of PBS and centrifuged again for 30 min at 100,000 × *g* and 4°C. Supernatant was removed using P1000 micropipette; pellets

were kept at 4°C covered with parafilm until all the pellets from subsequent centrifugations were obtained. Two MLA-130 rotors were used to perform the experiments discussed in this manuscript. After a batch of 10 samples was processed, one MLA-130 rotor was decontaminated with 4M guanidine thiocyanate for 1h at room temperature before another batch could be analyzed in the same rotor.

## PMCA Protocol

Three PTFE beads (Hoover Precision Bioproducts, http://www.hooverprecision.com) were placed inside 0.2 mL PCR tubes (Eppendorf, cat. no. 951010022) using tweezers. For the first PMCA round, the supplemented BH and the tubes with beads were taken into a biosafety level 3 (BSL3) facility where human prion experiments were performed. The sarkosyl precipitated prions were resuspended in 100 μL of 10% BH using a P100 micropipette and added into the 0.2 mL PCR tubes containing 3 PTFE beads. The samples were analyzed using Q700 sonicators (QSonica, cat. no. Q700A-110, https://www.sonicator.com), equipped with titanium microplate horns (QSonica, cat. no. 431MPXHTi) inside dry incubators set to 37°C. Between 220 and 250mL of distilled water was used to fill up the microplate horns. The sonication program included 30s of sonication, set at amplitude of 25–30, every 30 min. The first round of PMCA included 144 cycles (72h). For subsequent rounds, 90 μL of 10% BH were added to the 0.2 mL PCR tubes containing 3 beads and taken into the BLS3 laboratory. In there, 10 μL of the previous PMCA round were taken and added to the 90 μL of fresh substrate (100 μL final volume). The water in the horn was changed with fresh distilled water and the PCR tubes were sonicated for 30s every 30 min for 96 cycles (48h). When analyzing BC samples, a "pseudo-passage" of the first round was made, in which 90 μL of fresh substrate were added to 100μL from the first round. The purpose of this pseudo-passage was to reduce the viscosity of the solution by addition of more reaction mixture containing substrate.

## Proteinase K Digestion and Western Blotting

After PMCA, all samples (18 μL each) were digested using proteinase K (PK) at a concentration of 50 μg/mL and placed in an Eppendorf thermomixer at 37°C for 1h and 700 rpm agitation. PK digestion was stopped by boiling the sample at 100°C for 10 min after mixing with

NuPage (Invitrogen, cat. no. NP0007) or Novex (Invitrogen, cat. no. LC2676) sample loading buffer according to manufacturer's instructions. NuPage Bis-Tris (Invitrogen, cat. no. NP0342BOX) or Novex Tris-Glycine (Invitrogen, cat. no. XP00122BOX) gels were used with MES (Invitrogen, cat. no. NP0002) or Tris-SDS (Invitrogen, cat. no. LC2675) buffers, respectively. We additionally loaded $PrP^C$ (non–PK-digested brain homogenate) as an electrophoretic mobility control. Samples were run for 20 min at 70V and 1h and 40 min at 135V. Proteins were transferred onto nitrocellulose membranes (0.45 μm, Amersham, https://www.gelifesciences.com) for 1h at 800 mA. Then, membranes were blocked with 5% (wt/vol) dry nonfat milk in 0.05% (vol/vol) Tween 20 (prepared in PBS) for 1h at room temperature with shaking. Membranes were probed with monoclonal antibody 6D11 (BioLegend, cat. no. 808003, https://www.biolegend.com) at a dilution of 1:20,000 in 0.05% (vol/vol) Tween 20 (prepared in PBS) for 1 h at room temperature and washed in PBS-Tween (0.05%) 3 times (1 for 10 min and 2 for 5 min). Membranes we incubated for 1h at room temperature with secondary anti-mouse antibody (Sigma) used at 1:3,000 dilution in 0.05% (vol/vol) Tween 20 (prepared in PBS). Before developing, membranes were washed in PBS-Tween (0.05%) 3 times (1 for 10 min and 2 for 5 min). ECL chemioluminiscent reagent (Amersham) and a Chemidoc imaging system (BioRad, https://www.bio-rad.com) were used to develop and capture the images.

## Decontamination and Biosafety Procedures

In our laboratory, the basic decontamination procedures include incineration or chemical inactivation (2N NaOH, 4M guanidinium thiocyanate or 50% bleach for 1 hour). To decontaminate equipment and other nondisposable items we soaked, rinsed, or wiped the items with 2N NaOH solution and left it in contact for 1 hour. To decontaminate equipment that cannot be exposed to NaOH (e.g., sonicator horn, centrifuge rotors) we used 4M guanidinium thiocyanate. As general rule, we used 2N NaOH for plastic and glass items, and guanidinium thiocyanate for metal items. After decontamination, items were rinsed repeatedly with water. All spill cleanup material and surface decontamination waste was placed in a biohazard waste container designated for incineration. As a good working rule, we always use a disposable bench coat paper when performing an experiment using infectious samples. To work in the BSL3 laboratory, the use of disposable personal protective equipment (PPE) was mandatory.

Disposable PPE included 2 pairs of shoe covers, 2 gowns, 2 pairs of gloves, face mask, hair bonnet, and a face shield. All disposable PPE was discarded in biohazard waste containers designated for incineration. All the solid waste was disposed in plastic bags placed in cardboard boxes designated for incineration, which were sprayed with 2M NaOH (overnight drying), sprayed with 50% bleach (overnight drying), and then incinerated by the Environmental Health and Safety Department (EH&SD) of UTHealth. All the liquid waste was stored in plastic containers and adjusted to 2M NaOH final concentration. The plastic containers were sprayed with 2M NaOH (overnight drying), sprayed with 50% bleach (overnight drying), and disposed of by the EH&SD of UTHealth. All experimental procedures described here were done according to the standard operating procedure approved for our laboratory by the Biosafety Committee of UTHealth.
